# Access to COVID-19 Vaccines in High-, Middle-, and Low-Income Countries Hosting Clinical Trials

**DOI:** 10.1001/jamanetworkopen.2021.34233

**Published:** 2021-11-18

**Authors:** Reshma Ramachandran, Joseph S. Ross, Jennifer E. Miller

**Affiliations:** 1 National Clinician Scholars Program, Department of Internal Medicine, Yale School of Medicine, New Haven, Connecticut; 2 Veterans Affairs Connecticut Healthcare System, Yale University, West Haven, Connecticut; 3 Section of General Internal Medicine, Yale School of Medicine, New Haven, Connecticut; 4 Department of Health Policy and Management, Yale School of Public Health, New Haven, Connecticut; 5 Yale Program for Biomedical Ethics, Yale Center for Interdisciplinary Bioethics, and Bioethics International, New Haven, Connecticut

## Abstract

This cross-sectional study examines authorization and delivery of COVID-19 vaccines recommended by the World Health Organization in the countries where they were tested.

## Introduction

The COVID-19 pandemic has led to the rapid development of multiple vaccines, tested in clinical trials located in several countries.^1^ Low- and middle-income countries have experienced significant delays in vaccine access despite initiatives aiming to ensure fair distribution, such as COVID-19 Vaccines Global Access (COVAX).^2^ Because pharmaceuticals do not receive consistent and timely authorization for use in lower-income countries where they are tested,^3^ we examined authorization and delivery of COVID-19 vaccines recommended by the World Health Organization (WHO) in the countries where they were tested.

## Methods

For this cross-sectional study, we examined COVID-19 vaccines listed by the WHO for emergency use as of September 7, 2021. We then identified all completed clinical trials for these vaccines as of this date using the WHO COVID-19 Vaccine Tracker and Landscape and the McGill University COVID-19 Vaccine Tracker. We extracted trial primary completion dates, phase, and country locations from ClinicalTrials.gov. Each country was classified by national income group using the World Bank historical classifications for the year 2021.^4^ This cross-sectional study did not undergo institutional review board review and informed consent was not needed or sought because the study was not human participants research, in accordance with 45 CFR §46. It also followed the Strengthening the Reporting of Observational Studies in Epidemiology (STROBE) reporting guideline.

Through regional and national regulatory agency websites, we determined whether countries hosting vaccine clinical trials also authorized their use. Additionally, for each country, we extracted data on doses procured and delivered from the UNICEF COVID-19 Vaccine Market Dashboard and the Airfinity COVID-19 platform as of September 7, 2021. For each income group and manufacturer, we calculated the proportion of countries hosting clinical trials authorizing any vaccine tested in their population and the vaccines’ delivery. Last, we determined the median proportion of people 15 years and older who were able to receive a full vaccination series of the tested vaccine using population data from the United Nations’ World Population Dashboard. We conducted descriptive statistical analyses using Excel spreadsheet software version 16.0 (Microsoft).

## Results

Six unique COVID-19 vaccines were listed for emergency use by the WHO and tested in a total of 25 countries (Table 1) by September 7, 2021. Among 11 high-income countries hosting completed clinical trials, 10 (90.9%) authorized the tested vaccine and received enough doses to vaccinate a median 51.7% (IQR, 39.4%-76.7%) of their populations aged 15 years and older. Lower middle– and upper middle–income countries had high rates of authorization (100% and 90.9%, respectively), and median vaccination rates of 31.0% (IQR, 18.1%-37.6%) and 14.9% (IQR, 7.2%-48.6%), respectively. Examining ongoing and completed trials, corresponding rates were higher for high-income countries, but lower for all other income groups (Table 1).

**Table 1.** Access to Any COVID-19 Vaccine in Countries With Completed COVID-19 Vaccine Clinical Trials Categorized by National Income Group (as of September 7, 2021)

	No. (%)			Proportion of population fully vaccinated with doses delivered of tested vaccine, median (IQR), %^a^^,^^b^	Countries procuring doses of tested vaccine via COVAX, No. (median % delivered of procurement) [IQR]
	Countries that hosted COVID-19 vaccine clinical trials	Countries that authorized tested vaccine	Countries with doses delivered of tested vaccine		
Completed clinical trials					
All	25	23 (92.0)	23 (92.0)	39.4 (14.3-74.7)	8 (78.8) [36.9-99.8]
Low income	0	NA	NA	NA	NA
Lower middle income	3 (12.0)	3 (100.0)	3 (100.0)	31.0 (18.1-37.6)	1 (20.0)
Upper middle income	11 (44.0)	10 (90.9)	10 (90.9)	14.9 (7.2-48.6)	6 (98.7) [69.4-99.9]
High income	11 (44.0)	10 (90.9)	10 (90.9)	51.7 (39.4-76.7)	1 (38.0)
Ongoing and completed clinical trials					
All	37	35 (94.6)	35 (94.6)	39.5 (14.3-74.7)	16 (59.9) [36.9-99.0]
Low income	1 (2.7)	1 (100.0)	1 (100.0)	6.4	1 (15.4)
Lower middle income	6 (16.2)	6 (100.0)	6 (100.0)	20.2 (9.3-29.0)	5 (48.8) [20.0-98.7]
Upper middle income	12 (32.4)	10 (90.9)	10 (83.3)	14.6 (1.6-55.4)	6 (78.8) [55.3-99.2]
High income	18 (48.6)	18 (100.0)	18 (100.0)	67.9 (44.3-78.9)	4 (67.1) [54.4-80.8]

Abbreviations: COVAX, COVID-19 Vaccines Global Access; NA, not applicable.

^a^ Data at the country level for populations ages 15 to 64 years as well as 65 years and older were extracted from the World Population Dashboard hosted by the United Nations Population Fund. Data for populations ages 18 years and older as well as other specific age groups were unable to be extracted.

^b^ Data do not account for the population fully vaccinated with any COVID-19 vaccine, only the tested vaccine(s).

While Moderna completed clinical trials in 2 countries, AstraZeneca and Janssen completed trials in 14 and 10 countries, receiving authorization for their vaccines in 85.7% and 80.0% of these countries, respectively (Table 2). Across manufacturers, high-income countries received more doses to vaccinate larger median proportions of countries’ populations 15 years and older. COVAX delivered a median 15.4%, 48.8%, and 78.8% of procured doses of tested vaccines in low-, lower middle–, and upper middle–income countries that have hosted ongoing and completed trials, respectively.

**Table 2.** Access to COVID-19 Vaccines in Countries Hosting Completed COVID-19 Vaccine Clinical Trials Categorized by Manufacturer (as of September 7, 2021)

Manufacturer (vaccine)	Countries that hosted completed COVID-19 vaccine clinical trials, No.	No. (%)		No. (median proportion of population fully vaccinated) [range]^a^^,^^b^		
		Countries that authorized tested vaccine	Countries with doses delivered of tested vaccine	LMICs with completed trials	UMICs with completed trials	HICs with completed trials
Pfizer-BioNTech (BNT162b2)	9	9 (100.0)	8 (88.9)	0 (NA)	4 (15.3) [0.0-46.2]	5 (61.7) [51.2-66.5]
Moderna (mRNA-1273)	2	2 (100.0)	2 (100.0)	0 (NA)	0 (NA)	2 (43.3) [40.5-46.1]
Janssen–Johnson & Johnson (Ad26.COV2.S)	10	8 (80.0)	6 (60.0)	0 (NA)	6 (2.1) [0.0-7.4]	4 (3.3) [0.0-19.1]
AstraZeneca/Serum Institute of India (AZD1222/Vaxzevria/Covishield)^c^	14	12 (85.7)	11 (78.6)	1 (31.0)	7 (1.8) [0.0-30.4]	6 (9.4) [0.0-51.7]
Sinopharm (BBIBP-CorV)	6	6 (100.0)	6 (100.0)	1 (2.2)	3 (26.3) [14.3-46.4]	2 (38.8) [38.2-39.5]
Sinovac (CoronaVac)	5	5 (100.0)	5 (100.0)	1 (44.3)	3 (42.3) [28.7-47.3]	1 (69.7)

Abbreviations: HICs, high-income countries; LMICs, lower middle–income countries; NA, not applicable; UMICs, upper middle–income countries.

^a^ Estimate refers to the population within tested countries that is 15 years and older.

^b^ Data do not account for the population fully vaccinated with any COVID-19 vaccine, only the tested vaccine(s).

^c^ Because the same efficacy data were submitted for the vaccine Covishield, manufactured by the Serum Institute of India as the vaccine AZD1222 (Vaxzevria) by their common developers, Oxford University and AstraZeneca, this was considered to be a single vaccine for the analyses.

## Discussion

While countries of varying incomes have largely authorized the COVID-19 vaccines they helped test, high-income countries have received proportionately more doses, enabling them to more fully vaccinate their populations. Including low- and middle-income countries in research can be an important goal; however, inclusion should correspond with fair access to research benefits, to help avoid exploitation. These wealth-based access inequities among countries hosting trials parallel general disparities in COVID-19 vaccine access, as high-income countries have successfully procured and administered doses ahead of low- and middle-income countries.^5,6^

Study limitations include inability to determine the number of enrolled trial participants per country, which was not systematically reported, and the impact of manufacturing errors and safety concerns on dosage delivery, particularly affecting AstraZeneca and Janssen. Further, we focused on vaccination rates with vaccines tested in clinical trials hosted by each country, not overall vaccination rates with any authorized COVID-19 vaccine.
